# Chicago as a Medical Centre

**Published:** 1885-11

**Authors:** 


### CHICAGO AS A MEDICAL CENTER.

A well-known statistician recently made the assertion that the total number of matriculates at the various Chicago medical schools, regular and irregular, exceeded that of any other city in the United States. We are not in possession of the facts necessary to corroborate this statement. It is certainly true, however, that the number of medical students in Chicago is considerably increasing. The facilities for clinical instruction are rapidly multiplying.

The completion of ST. LUKE'S HOSPITAL has added materially to the resources of the CHICAGO MEDICAL COLLEGE. The PRESBYTERIAN HOSPITAL, now in process of construction, will prove an invaluable adjunct to the RUSH MEDICAL COLLEGE. Rooms in the handsome edifice of the COLLEGE OF PHYSICIANS AND SURGEONS have been appropriated for the use of patients, and we are reliably informed, plans for a new hospital, to be attached to this institution, are in the architects' hands. A hospital for women has been recently built on the West Side, and a Lying-in Hospital is in process of construction on the South Side.

Under the operation of the new Anatomical Act, it is reasonable to expect that material for dissection and the study of gross pathology will be abundant.

We have, however, no physiological laboratories, and no facilities for experimental research. Until this want is supplied, Eastern medical schools will retain their acknowledged superiority.

